# The future of fish‐based ecological assessment of European rivers: from traditional EU Water Framework Directive compliant methods to eDNA metabarcoding‐based approaches

**DOI:** 10.1111/jfb.14176

**Published:** 2019-11-18

**Authors:** Didier Pont, Alice Valentini, Mathieu Rocle, Anthony Maire, Olivier Delaigue, Pauline Jean, Tony Dejean

**Affiliations:** ^1^ Institute of Hydrobiology and Aquatic Ecosystem Management University of Natural Resources and Life Sciences, Gregor‐Mendel‐Strasse 33 Vienna Austria; ^2^ SPYGEN, 17 rue du Lac Saint‐André, Savoie Technolac Le Bourget du Lac France; ^3^ Compagnie Nationale du Rhône Lyon France; ^4^ EDF R&D LNHE ‐ Laboratoire National d'Hydraulique et Environnement Chatou France; ^5^ IRSTEA, HYCAR Research Unit Antony France

**Keywords:** eDNA, fish‐based assessment methods, rivers, Water Framework Directive, WFD

## Abstract

Most of the present EU Water Framework Directive (WFD) compliant fish‐based assessment methods of European rivers are multi‐metric indices computed from traditional electrofishing (TEF) samples, but this method has known shortcomings, especially in large rivers. The probability of detecting rare species remains limited, which can alter the sensitivity of the indices. In recent years, environmental (e)DNA metabarcoding techniques have progressed sufficiently to allow applications in various ecological domains as well as eDNA‐based ecological assessment methods. A review of the 25 current WFD‐compliant methods for river fish shows that 81% of the metrics used in these methods are expressed in richness or relative abundance and thus compatible with eDNA samples. However, more than half of the member states' methods include at least one metric related to age or size structure and would have to adapt their current fish index if reliant solely on eDNA‐derived information. Most trait‐based metrics expressed in richness are higher when computed from eDNA than when computed from TEF samples. Comparable values are obtained only when the TEF sampling effort increases. Depending on the species trait considered, most trait‐based metrics expressed in relative abundance are significantly higher for eDNA than for TEF samples or *vice versa* due to over‐estimation of sub‐surface species or under‐estimation of benthic and rare species by TEF sampling, respectively. An existing predictive fish index, adapted to make it compatible with eDNA data, delivers an ecological assessment comparable with the current approved method for 22 of the 25 sites tested. Its associated uncertainty is lower than that of current fish indices. Recommendations for the development of future fish eDNA‐based indices and the associated eDNA water sampling strategy are discussed.

## 1 INTRODUCTION

In European rivers, phytobenthos, aquatic plants, macroinvertebrates and fish are the biological quality elements (BQE) that are monitored to meet the requirements of the EU Water Framework Directive (WFD; EC, 2000). Since 2000, a very large number of ecological assessment methods have been developed and implemented in different EU member states (Birk *et al*., 2012). One of the main common characteristics of these methods is the use of the “reference condition approach” (Bailey *et al*., 1998), in which the observed species assemblage in a given water body is compared with the assemblage expected in a reference situation with the same environmental characteristics but unexposed to any human‐induced stress (Hughes *et al*., 1998). The final ecological quality ratio (EQR) is the score synthetising the comparison between observed and expected BQEs (Jones *et al*., 2010). Each country has developed its own methods for each of the four BQEs and the final implementation of each of these methods was possible only after an intercalibration exercise performed to ensure the comparability of ecological quality class limits between EU member states (Birk *et al*., 2013; Poikane *et al*., 2014).

Fish communities respond to almost all types of anthropogenic disturbances, including degradation of water quality, alteration of the hydromorphological habitat and connectivity disruption at a large scale (Ormerod, 2003), which is consistent with the scale of the spatial unit of watercourse management, from a few km to a few dozen km long. Since the 1970s, various fish‐based biotic indices have been widely used to assess the ecological quality of rivers and most of them are based on the index of biotic integrity (IBI; Karr, 1981). This index uses the reference condition approach and is also the first description of a multimetric index using a series of metrics based primarily on assemblage structure and function. Each measurement is scored according to baseline conditions and integrated into a scaled numerical index to reflect the ecological health of the species assemblage (Fausch *et al*., 1990; Simon, 1999). Because of their characteristics, this family of indices corresponds to the requirements of the WFD and most of the fish indices implemented and intercalibrated in Europe belong to this family of assessment methods (Pont, 2011). Unlike phytobenthos or macroinvertebrates, fish species can be identified quickly. Conversely, electrofishing, the sampling method officially recommended for river fish by the WFD (CEN, 2003), requires a large and qualified staff and is expensive, at a time when the costs of the assessment are under scrutiny but its quality be maintained (Borja & Elliott, 2013). Furthermore, electrofishing cannot be performed in low‐conductivity waters (Allard *et al*., 2014).

Electrofishing is an efficient sampling method but has well‐known shortcomings. Due to sampling difficulties, the probability of detecting rare species (*i.e*., those representing <1% of the total abundance; Paller, 1995), remains limited (MacKenzie *et al*., 2015). In wadable streams, correct evaluation of species richness would imply either sampling a length of more than 27 times the stream width in a single pass or increasing the number of passes (Daulwater & Pert, 2003; Fischer & Paukert, 2009; Vehanen *et al*., 2013). Subsequently, the minimum sampled reach length required by the EU standard sampling protocol (CEN, 2003; FAME, 2005) of 10 to 20 times the river width guarantees the capture of only the most common species (Paller, 1995).

In large rivers, no one type of gear was found to be appropriate for quantitative sampling in all existing habitats (Casselman *et al*., 1990; Goffaux *et al*., 2005; Zajicek & Wolter, 2018). In wadable rivers, electrofishing is the standard fishing method. But if the rivers are large and deep, it is restricted to the shallow littoral shoreline (CEN, 2003), which implies an overestimation of sub‐surface species at the expense of mid‐channel and benthic species (Eros *et al*., 2017; Pont *et al*., 2018; Zajicek & Wolter, 2018). Electrofishing is currently considered the most appropriate method, but in practice, a combination of several types of gear is required to correctly estimate the total species richness (Casselman *et al*., 1990; Zajicek et Wolter, 2018), which greatly increases the cost of monitoring.

Fish‐based ecological assessments of rivers are very sensitive to sampling effort (Cao *et al*., 2001). The contribution of rare taxa to aquatic bioassessments remains a subject of controversy among researchers (Guareschi *et al*., 2017; Poos & Jackson, 2012). Metrics based on the relative abundance of fish taxa are less sensitive to the loss of rare taxa than richness‐based metrics and the removal of all fish that occur as singletons or doubletons significantly affects the IBI metrics and final score (Wan *et al*., 2010). The sensitivity of the IBI to rare species seems to increase with species richness and a greater sampling effort is thus needed in large rivers (Dolph *et al*., 2010). However, rare species are very often sensitive species and potentially of great interest for bioassessment.

Since its first application to macro‐organisms, environmental (e)DNA has increasingly appeared to be a promising non‐invasive method for improving aquatic biomonitoring (Lawson Handley, 2015). With the emergence of next‐generation sequencing platforms and the use of universal PCR primers (eDNA metabarcoding), large collections of taxa can now be identified with a single analysis (Taberlet *et al*., 2012). This not only offers the possibility to detect rare or elusive species but also allows the rapid assessment of complete fish communities (Bohmann *et al*., 2014; Thomsen *et al*., 2012; Valentini *et al*., 2016). In rivers, fish DNA is collected from water samples and, unlike the DNA of small planktonic organisms (*e.g*., diatoms), is only extra‐organismal DNA (cellular or extra‐cellular) and degraded (Taberlet *et al*., 2012). Recent studies have shown that eDNA metabarcoding is a very efficient method for monitoring fish communities in rivers (Civade *et al*., 2016; Yamanaka & Minamoto, 2016; Nakagawa *et al*., 2018; Pont *et al*., 2018).

By taking large volumes of water (30–60 l) and using short genetic markers (fewer than 100 bp), the species richness estimated from eDNA in a stream and a large river was on average 1.1–1.31 and 1.7–3.5 times higher, respectively, than that detected by traditional electrofishing (Civade *et al*., 2016; Pont *et al*., 2018). In both cases, with a single eDNA sampling session, the species richness was comparable to the cumulative number of species collected during long‐term electrofishing surveys. Furthermore, some studies have shown that species relative abundances are correctly estimated with the eDNA approach (Li *et al*., 2019; Pont *et al*., 2018; Thomsen *et al*., 2012). Nevertheless, investigating absolute density or biomass as well as the relative abundance of age or size classes remains a great challenge for eDNA metabarcoding approaches.

The potential use of eDNA in future methods of ecological assessment of aquatic environments has already been specifically mentioned in the context of the WFD (Cordier *et al*., 2017; Hering *et al*., 2018; Pawlowski *et al*., 2018). Environmental DNA appears to be a well‐suited sampling approach for fish due to its suitability for taxonomical identification at the species level (Hering *et al*., 2018). However, a fish eDNA‐based assessment method (in contrast to a method for diatoms and invertebrates) will require a completely new sampling procedure in addition to a shift from morphology‐based to eDNA‐based taxonomic description. In addition, the spatio‐temporal representativeness of an eDNA water sample is different from that of a traditional electrofishing sample: eDNA integrates a larger extent of the river reach, including the deepest part of the watercourse section but also from several hundred m to several tens of km upstream from the sampling point, depending on the characteristics of the river (Deiner *et al*., 2016; Pont *et al*., 2018).

Our main objective is to evaluate the possibilities of adapting the current WFD‐compliant fish‐based river assessment methods to eDNA‐based methods. To meet this objective, the following points are considered: (1) a review of the 25 current WFD‐compliant fish‐based river assessment methods to determine the extent to which these methods can be directly applied to eDNA data (types of metrics and reference condition approach); (2) an analysis of the representativeness of eDNA‐based metrics based on a comparison of the values of the most commonly used species trait‐based metrics computed from both a traditional electrofishing (TEF) survey and an eDNA survey performed in the same river sections (RS) and at the same period in a large river (Rhône River, France); (3) a test of the influence of TEF sampling effort on the estimation of the species trait‐based metrics; (4) an adaptation of an existing fish index to eDNA data and a test of its adequation *v*. the current ecological assessment of the corresponding water bodies; (5) an evaluation of the uncertainty associated with this adapted fish index and its seasonal variability. Finally, recommendations for the development of fish eDNA‐based indices compliant with the WFD are proposed and discussed.

## 2 MATERIALS AND METHODS

### 2.1 Review of WFD‐compliant national methods

The 25 national fish‐based river assessment methods considered in this paper were part of the official reporting procedure of the European intercalibration exercise to ensure compliance with the WFD requirements and that good ecological status represents the same level of ecological quality everywhere in Europe (Birk *et al*., 2012). Eighteen EU member states joined the intercalibration process from 2008 to 2011 (Pont, 2011): Austria (AT), Belgium–Flanders (BF), Belgium–Wallonia (BW), Czech Republic (CZ), Germany (DE), England–Wales (EN‐WA), Finland (FI), France (FR), Ireland (IR), Lithuania (LT), Netherlands (NL), Portugal (PT), Romania (RO), Scotland (SC), Slovenia (SL), Spain (SP), Slovakia (SV), Sweden (SW). Seven additional countries joined afterwards: Bulgaria (BU), Denmark (DN), Greece (GR), Hungary (HU), Italy (IT), Latvia (LV), Poland (PL). The methods were analysed according to three criteria: (a) the concept of the method (*e.g*., mono‐ versus multi‐metric, classical or predictive IBI and taxonomy versus functional trait‐based metrics), (b) the evaluation of the reference conditions (*e.g*., dataset, expert opinion, historical data and modelling) and (c) the different types of metrics following the classification of Birk *et al*. (2012).

### 2.2 Traditional electrofishing

Regular TEF surveys were performed in spring, summer and autumn each year by Electricité de France (EDF) for 40 years in several river stretches (RS) situated in the main channel of the Rhône River (Maire *et al*., 2019). Fish were sampled from a boat along the banks with only one pass, as recommended for large and non‐wadable rivers (FAME, 2005). Depending on the operator, fish were sampled at two to three neighbouring sites either by 30‐min effective continuous sampling per site or by point abundance sampling (Maire *et al*., 2019) equivalent to 15 min effective continuous sampling for 20 point sampling units per site (Pont *et al*., 1992).

**FIGURE 1:**
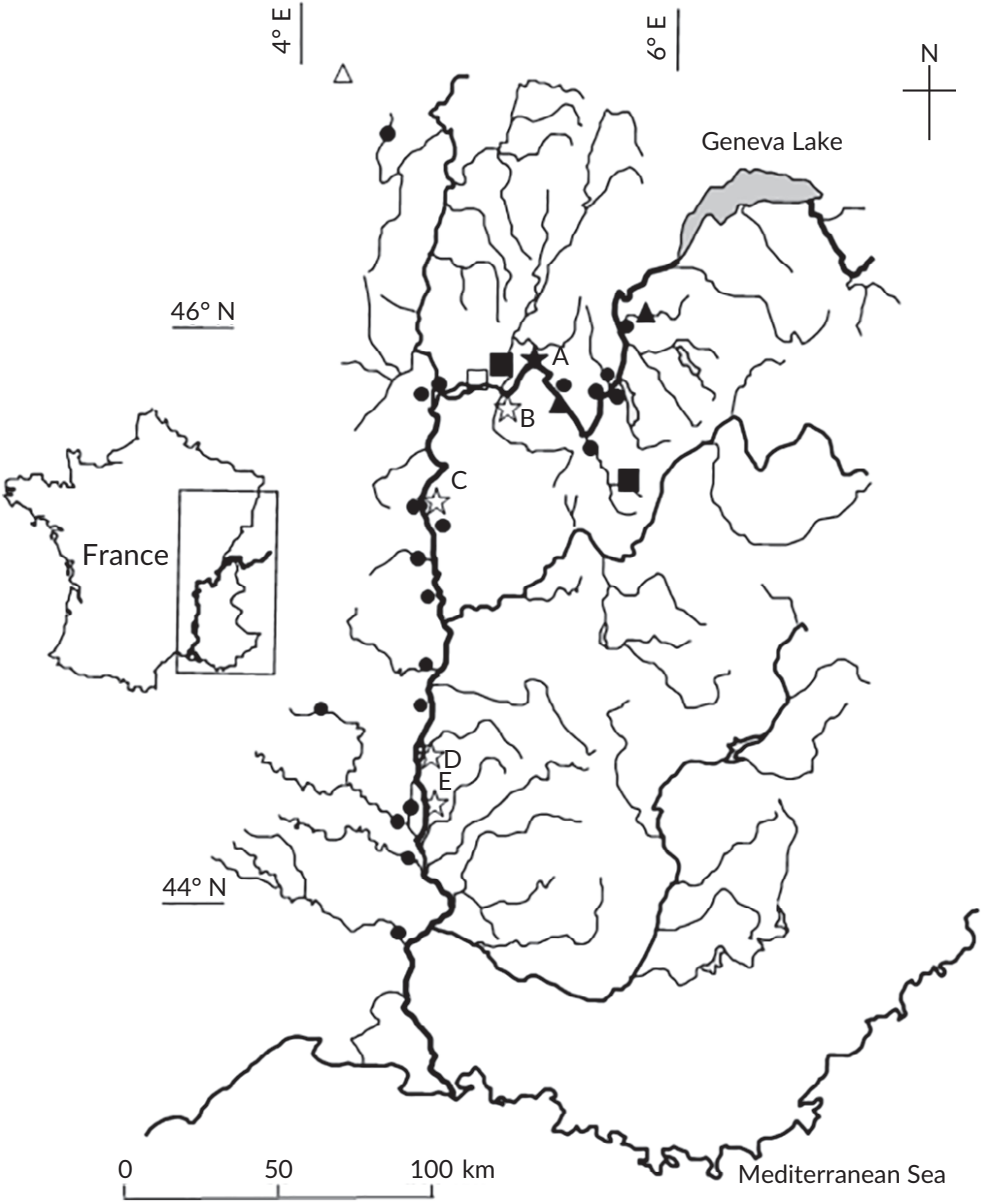
Sampling locations along river stretches (RS) A to E (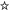) of the main channel of the Rhône River, France, using both traditional electro‐fishing (TEF) and eDNA. 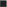, Sites sampled every 2 months (September 2015– August 2016); 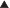, sites where ten eDNA water samples (filtration capsules) were collected once; 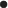, sites located on the tributaries or the Rhône River itself sampled once for eDNA. The 10 metric fish index and the adapted six‐metric fish index were computed at all sites with a black filled symbol within natural water bodies

Two RSs were located in the Upper Rhône along one of the last free‐flowing sections (A and B) and three others (C, D and E), within impounded sections of the Lower Rhône (Figure 1). The maximal distance between TEF sites in a given RS was <2 km, except for RS D (6 km). The results from a generalised linear model demonstrated that species richness did not have any consistent temporal trend during the 2 year period before the eDNA survey (summer 2013 to spring 2016: six sampling campaigns). The mean sampling effort per RS and per sampling campaign was 103, 92, 40, 77 and 78 min for RSs A, B, C, D and E, respectively. This effort was comparable to that in the official WFD monitoring survey: 75 to 100 point sampling units; 68–90 min (Tomanova *et al*., 2013). Individuals were identified to the species level following the taxonomic nomenclature of Keith *et al*. (2011).

### 2.3 eDNA sampling sites and processing method

Environmental DNA sampling was performed using a filtration device (VigiBOAT, SPYGEN; www.spygen.com) including a peristaltic pump (nominal flow of 1.1 l min^−1^) or directly with a peristaltic pump. In both cases, the water was filtered through a VigiDNA 0.45 μM crossflow filtration capsule (SPYGEN), with disposable sterile tubing for each filtration capsule. Each filtration took 30 min for a water volume of *c*. 30 l. At the end of each filtration, the water inside the capsule was emptied and the capsule was refilled with 80 ml of CL1 conservation buffer (SPYGEN) to avoid eDNA degradation and stored at room temperature (Pont *et al*., 2018). This sampling protocol was similar for all studied sites (Figure 1), but the number of water samples (and corresponding filtration capsules and eDNA analyses) varied.

Environmental DNA water samples were collected in April–May 2016 (2 filtration capsules per site) along the Rhône River from Geneva Lake to the Mediterranean Sea (see Pont *et al*., 2018 for details). Environmental DNA samples were taken at the same location as TEF samples in April–May 2016 at RS B and RS D, 7 km upstream at RS A and RS C and 12 km downstream at RS E. In the last case, the distance was relatively long, but RS E was located in a 50 km long artificial concrete channel with homogeneous hydro‐morphological characteristics (Olivier *et al*., 2009). Eighteen natural water bodies classified as “natural” from the Rhône River or tributaries (1 km to a few km upstream from the confluence) were also sampled during the same survey (Figure 1) with a similar sampling effort (2 filtration capsules per site). Environmental DNA was also sampled six times from September 2015 to August 2016 (Milhau *et al*., 2019) at three sites (three filtration capsules per sampling date) located on the Tier River, the Ain River and the upper Rhône River at Jons (river widths of 9, 80 and 150 m, respectively). Ten water samples were also collected once at 3 sites on the Rhins River, the Usses River and the upper Rhône River at Brangues, with river widths of 10, 11 and 95 m, respectively. Finally, three sites were sampled once with three filtrations (the Charbonnières, Dorne and Azergues Rivers).

The complete eDNA processing method and the marker reference database are described in Pont *et al*. (2018) and Valentini *et al*. (2016). In brief, after eDNA extraction from the conservation buffer, twelve 50 cycle PCR amplifications per filtration capsule extract were performed. After purification, the PCR products were pooled in equal volumes to achieve a theoretical sequencing depth of 500,000 reads per sample. The libraries were prepared using the MetaFast protocol (Fasteris; www.fasteris.com) and paired‐end sequencing (2 × 125 bp) was carried out on an Illumina HiSeq 2500 sequencer (www.illumina.com). Sixteen negative extraction controls and seventeen negative PCR controls (ultrapure water, 12 replicates) were amplified and sequenced in parallel to the samples to monitor possible contaminants.

The marker reference database used for molecular operational taxonomical unit identification (MOTU) included almost all French freshwater fish species (Valentini *et al*., 2016). The molecular markers did not discriminate between species belonging to different genera for two groups, which were referred to as Cypr_1 (*Telestes souffia* (Risso 1827), *Chondrostoma nasus* (L. 1758) and *Parachondrostoma toxostoma* (Vallot 1837)) and Cypr_2 (*Hypophthalmichthys molitrix* (Valenciennes 1844) and *Ctenopharyngodon idella* (Valenciennes 1844)). Species in some genera were not differentiated: *Salvelinus alpinus* (L. 1758) and *Salvelinus fontinalis* (Mitchill 1814) (Sal_spp); *Leuciscus idus* (L. 1758) and *Leuciscus leuciscus* (L. 1758) (Leu_spp); *Carassius carassius* (L. 1758), *Carassius auratus* (L. 1758) and *Carassius gibelio* (Bloch 1782) (Cas_spp); *Alosa fallax* (Lacépède 1803) and *Alosa alosa* (L. 1758) (Alo_spp); *Cottus gobio* L 1758 and *Cottus petiti* Bǎcescu & Bǎcescu‐Meşter 1964 (Cot_sp); and *Lampetra planeri* (Bloch 1784) and *Lampetra fluviatilis* (L. 1758) (Lam_spp).

Four MOTUs were discarded from our analyses. Cypr_2 corresponded only to farmed species (Keith *et al*., 2011) and their detection in the Rhône River can only be due to effluent from fish ponds. The abundance of DNA copies from *Oncorhynchus mykiss* (Walbaum 1792), a commonly farmed species, was probably related to the release of individuals by fishery associations at the time of the eDNA sampling campaign, a few weeks after the opening of the fishing season. *Coregonus lavaretus* (L. 1758) (MOTU Cor_spp), a typical lake‐dwelling species, is very rare in the upper Rhone River and the abundance of its eDNA in the upper Rhone River was mainly due to downstream transport from Lakes Geneva and Bourget (Pont *et al*., 2018). The case of *S. alpinus* was similar, as it was also abundant in the same upstream lakes (Keith *et al*., 2011).

### 2.4 Representativeness of the eDNA‐based species trait metrics

We focused on species trait‐based metrics because they are the most commonly used among current WFD‐compliant national methods (Pont *et al*., 2011). Each species was assigned to a species trait category according to previous classifications (Logez *et al*., 2013; Marzin *et al*., 2014; Noble *et al*., 2007). Among the most common species trait‐based metrics currently used, we selected the nine most dissimilar metrics; *i.e*., metrics based on species trait category lists with fewer than 50% of species in common between each pair of metrics: benthic feeding species (BEN), eurytopic (generalist) species (EUR), insectivorous species (INS), omnivorous species (OMN), phytophilic species (PHY), potamodromous species (POT; migratory movements in freshwater habitats), rheophilic species (RHE), tolerant to pollution species (TOL) and pelagic species (PEL).

For MOTUs that could not be identified at the species level, the assignment to one category was evident when all species had the same functional profile (Car_spp and Cot_sp). When one species of the MOTU was clearly the most abundant in the TEF dataset (at least 95% of the fish caught for the species belonging to the considered MOTU), the ecological profile of this species was assigned to the MOTU *C. nasus* for Cypr_1 and *L. leuciscus* for Leu_spp. Sal_spp and Cypr_2 were not included in the analyses (see above for explanation). Each metric was considered either in absolute number of species or in relative number of MOTUs and computed for all the eDNA water samples.

The values of these trait‐based metrics computed from eDNA water samples were compared with values obtained from TEF samples from the same period (spring 2016). To test the influence of TEF sampling effort, the eDNA metrics were tested against TEF metrics computed from three (summer 2015 to spring 2016) and six (summer 2014 to spring 2016) sampling sessions. For species richness and each of the richness‐based metrics, generalised linear models (GLM) were used to test the significance of the difference between the two methods after considering the RS effect (MASS package, glm function, Poisson distribution in R; Venables & Ripley, 2002; R Core Team, 2018; www.r-project.org). The model's residual deviance was used as the goodness‐of‐fit criterion for model evaluation and comparison. For metrics based on the relative number of individuals, negative binomial models were used (R software, MASS package, glm.nb function) with an offset (logarithm of the total number of individuals) to control for sample size.

### 2.5 Comparability of a fish eDNA‐based assessment method with the current approach

The assessments resulting from an eDNA‐based method were compared with the current water assessment using a subset of 25 of all our eDNA sites belonging to natural water bodies (*i.e*., excluding sites located in heavily modified water bodies). These sites covered a wide range of environmental conditions (river width: 4–163 m, river slope: 0.07–44 ‰, upstream catchment area: 28–74 447 km^2^ and altitude: 3–598 m a.m.s.l.). When more than 2 eDNA water samples were collected per site, only two of them were randomly selected to standardise the eDNA sampling effort.

A predictive multimetric fish index developed to assess French river water bodies (Pont *et al*., 2006; Logez & Pont, 2011; Marzin *et al*., 2014) was adapted to evaluate the ecological quality of water bodies from eDNA samples. This index was composed of ten trait‐based metrics (functional metrics) and one additional metric reflecting the strength of the young in the year class for brown trout *Salmo trutta* L. 1758 (Logez & Pont, 2011). The latter metric was used only for the *S. trutta* and *Thymallus thymallus* (L 1758) zones. For each river site, the observed values of these metrics were compared with theoretical values computed from a set of models predicting the functional composition of the fish assemblage in the quasi‐absence of anthropogenic disturbance (least‐disturbed reference conditions; Stoddard *et al*., 2006). At each site, theoretical metrics were modelled as a function of eight environmental descriptors (upstream catchment area, river slope, river width, mean annual air temperature at the site, mean annual air temperature and rainfall over the upstream catchment area, hydrological regime and dominant sediment over the catchment) known to influence fish assemblages (Pont *et al*., 2005; Pont *et al*., 2006). Values of environmental variables were derived from the French National Institute of Geographic and Forest Information (www.geoportail.gouv.fr) and the French Meteorological Institute (www.mfi.f; Vidal *et al*., 2010). The EQR metrics (normalised distance between observed and theoretical metric values; Marzin *et al*., 2014) showing the lowest values were aggregated into an EQR fish index varying from 0 to 1. The sites were assigned to one of the five ecological status classes according to threshold values of the EQR fish index defined in agreement with the EU intercalibration rules (EC, 2011).

The *S. trutta* age class metric was first removed from this existing index as eDNA samples provide no information about the age or size‐class distribution (eDNA‐10FI, eDNA‐based 10‐metric fish index). Four of the ten remaining functional metrics were expressed in number of species (*i.e*., limnophilic reproduction habitat, stenothermal, omnivorous and tolerant species), three in relative number of species (*i.e*., limnophilic, oxyphilous and intolerant species) and three in relative number of individuals (*i.e*., oxyphilous species, habitat degradation intolerant and running water spawning habitat). The fish index was computed first with these ten functional metrics from our fish‐eDNA samples. Based on our previous study (Pont *et al*., 2018), we hypothesised that the metrics expressed in number of species would be the most different between the eDNA and conventional approaches. Then, we computed an adapted version of the fish index (eDNA‐6FI, eDNA‐based six‐metric fish index) based only on the six metrics expressed in relative abundance of species or individuals.

Environmental DNA‐10FI and eDNA‐6FI were computed at 25 sites located in natural water bodies of the Rhône River itself (6 sites) or in tributaries (19 sites). The ecological status of the sites obtained from these two fish indices was compared with the official ecological status of the corresponding natural water bodies evaluated by the Rhône‐Mediterranean‐Corsica Water Agency (assessment results available at http://sierm.eaurmc.fr/surveillance/eaux-superficielles/index.php). As sites classified as having a high, poor or bad status were rare, we distinguished only between good and degraded ecological statuses (high–good against moderate–poor–bad ecological classes). The significance of the association between the two categorical variables was tested using a *χ* ^2^‐test.

The spatially‐driven uncertainty of eDNA‐6FI was evaluated (CV) in three sites (Haut‐Rhône at Brangues, Rhins and Usses) where ten eDNA water samples were collected once. At each site, eDNA‐6FI was computed for each of the 45 possible combinations of two samples. The seasonal variability of eDNA‐6FI was estimated from eDNA samples collected every 2 months 2015–2016 from the Tier River, the Ain River and the Upper Rhône at Jons.

## 3 RESULTS

### 3.1 WFD‐compliant national methods

Only the three closely related methods from EN‐WA, SC and IR were not multi‐metric indices, resulting from the comparison between observed species abundances and predicted species abundances under reference conditions (SNIFFER, 2011). Sixteen methods were national adaptations of the original IBI from Karr (1981); *i.e*., multimetric indices: AT, BF, BU, BW, CZ, DE, DN, FI, HU, IT, LT, NL, PT, SL, SP, SV. Six national methods (FR, GR, LV, PL, RO, SW) were multi‐metric predictive indices where the reference condition for each metric was predicted as a function of local environmental conditions in the quasi‐absence of human disturbance (Oberdorff *et al*., 2002; Pont *et al*., 2006).

For 14 of the 25 member states, fish indices were scored according to reference conditions based on combined examination of actual data from minimally to least‐affected sites (Stoddart *et al*., 2006), historical data and expert judgement. The other member states calibrated their indices or metrics using a reference dataset of minimally affected sites (BF, EN, FR, GR, HU, IR, LV, PL, RO, SC, SW). Among the 198 different metrics considered in these 25 methods (Table 1), 161 were expressed in number of species, relative abundance or relative biomass (38.4%, 42.4% and 0.5% of the total, respectively), whereas 34 metrics were expressed in absolute density or biomass (14.1% and 3.0%, respectively). Only three metrics (1.52%) were established by expert judgement.

**TABLE 1:** Classification of the 198 metrics from the 25 national fish‐based river assessment methods subjected to the European intercalibration process. Metrics are classified according to their types and the unit in which they are expressed. Metrics belonging to types and expressed in units allowing calculation from eDNA data are in bold letters

Metric Types	Metric Units						
	Species richness	Relative abundance	Relative biomass	Density	Biomass	Expert judgment
Species trait‐based metrics						
Habitat guilds	**19**	**12**	**1**	2		
Migratory guilds	**5**	**4**		1		
Perturbation tolerance	**13**	**15**		1		
Reproductive guilds	**14**	**13**				
Trophic groups	**8**	**10**		5		
Other metrics						
Whole assemblage	**2**	**7**		2	4	
Taxonomy‐based metrics	**9**	**6**		8	2	
Biogeographical status	**5**	**5**		1		
Health alteration		2				
Length or age class	1	10		8		3

Most of the metrics were trait‐based (*n =* 123 metrics, 62.1% of the total). Taxonomy‐based metrics (*n =* 25, 12.6% of the total, mainly river‐type indicator species) were considered by 13 member states and 15 metrics (7.6% of the total) were related to the structure of the fish assemblage (*e.g*., total density, total biomass and diversity). Only 10 metrics (5.1% of the total) described the age or size structure of indicator species populations. But 12 of the 143 trait‐based metrics also considered age or size criteria. In total, at least one metric related to age or size structure was considered by 14 of the 25 member states. Eleven metrics (5.5% of the total) from 8 national methods are related to the number of alien or native species. The health status of individuals (injuries, anomalies and parasites) was only considered by two national methods. Among the 123 trait‐based metrics, the frequencies of metrics based on habitat guilds, reproductive guilds, trophic groups and tolerance to perturbation were comparable whereas migratory guilds were less frequently used: 18.7% to 27.6% and 8.1% of the total number of trait‐based metrics, respectively.

### 3.2 Representativeness of eDNA‐based metrics

Among the 33 detected MOTUs corresponding to morphologically defined species, only Lam_spp and *T. thymallus* were not caught by TEF in the five RSs (Table 2). Furthermore, *L. planeri* and *T. thymallus* were recently caught by TEF upstream from RS BUGA (Pont *et al*., 2018).

**TABLE 2:** Relative per cent abundance of species molecular operational taxonomical unit identification (MOTU) detected from eDNA metabarcoding of water samples and of species caught by traditional electrofishing (TEF) in river stretches (RS) A–E

Species names	MOTU	Metrics	RS A		RS B		RS C		RS D		RS E	
			TEF	eDNA	TEF	eDNA	TEF	eDNA	TEF	eDNA	TEF	eDNA
*Abramis brama*	Abr_bra	BEN, OMN, TOL		3.61		1.42	2.25	6.34	0.12	14.48	0.07	13.86
*Alburnus alburnus*	Alb_alb	PEL, EUR INS TOL	12.82	2.02	1.09	1.57	16.53	10.80	57.14	4.15	69.81	5.98
*Alburnoides bipunctatus*	Alb_bip	PEL RHE, INS	41.59	0.81	64.91	5.65	0.09	5.35	1.59	3.67	0.56	1.14
*Alosa fallax*	Alo_spp	PEL, RHE							0.01		0.01	
*Ameiurus melas*	Ame_mel	BEN, INS, TOL		0.41			1.49					
*Anguilla anguilla*	Ang_ang	BEN, EUR, TOL					0.03		1.11	0.53	1.96	0.78
*Barbus barbus*	Bar_bab	BEN, RHE, OMN, POT	5.91	19.14	13.18	23.23	2.54	13.92	2.01	11.08	2.68	10.43
*Barbatula barbatula*	Bar_bar	BEN, RHE, INS	0.50	6.07	0.04	4.88	0.08	1.56	4.14	3.92	0.03	3.71
*Blicca bjoerkna*	Bli_bjo	BEN, OMN, TOL	1.93	2.10	0.33	2.40	21.37	3.96	6.24	8.07	2.98	3.91
*Cottus gobio*	Cot_sp	BEN, EUR, INS	0.56	6.03	0.33	13.15		3.39	0.08	0.72		0.60
*Cyprinus carpio*	Cyp_car	BEN, OMN, PHY, TOL	0.06	0.17	0.01	0.11	0.36	0.73	0.05	0.84	0.06	0.58
*Esox lucius*	Eso_luc	PEL, PHY, POT	0.18	3.62		2.27	0.01	0.30	0.03	0.22	0.03	0.03
*Gasterosteus aculeatus*	Gas_acu	PEL, EUR, INS, PHY, TOL	0.10	0.38	0.04	0.41	0.04	0.02	0.10	0.13		0.02
*Gobio gobio*	Gob_gob	BEN, EUR, OMN	7.86	11.12	1.16	6.83	8.76	2.85	7.25	5.98	1.44	3.90
*Gymnocephalus cernuus*	Gym_cern	BEN, INS,TOL		4.11		3.21	0.24	10.62		5.22		3.31
*Lampetra planeri*	Lam_spp	BEN, RHE		0.04		0.22		0.01				
*Lepomis gibbosus*	Lep_gib	PEL, INS, TOL	0.04			0.38	0.79	1.27	0.03	0.15	0.37	0.17
*Perca fluviatilis*	Perc_flu	PEL, TOL	0.04	0.74	0.13	0.38	0.43	1.13	0.12	0.53	0.10	0.42
*Phoxinus phoxinus*	Pho_pho	PEL, EUR, INS	0.28	2.11	0.07	2.16	0.11	0.77	0.06	1.65	0.01	0.53
*Pseudorasbora parva*	Pse_par	PEL, OMN, TOL	0.08	0.06	0.05	0.10	4.29	0.47	1.87	0.54	1.68	0.42
*Rhodeus sericeus*	Rho_ser	PEL	0.02	0.13	0.07	0.14	5.64	0.46	2.16	0.67	3.21	0.76
*Rutilus rutilus*	Rut_rut	PEL, OMN, TOL	1.47	6.18	0.34	3.80	8.94	18.22	0.82	19.58	2.17	36.03
*Salaria fluviatilis*	Sal_flu	BEN, EUR, INS		0.75	0.22	1.02	0.17	0.47	0.03	0.42	0.03	1.05
*Salmo trutta*	Sal_tru	PEL, EUR, POT	0.12	5.27	0.02	5.44		0.49	0.08	1.00	0.04	1.54
*Sander lucioperca*	San_luc	PEL, TOL		1.94		0.39	0.04	3.52		0.51	0.04	0.50
*Scardinius erythrophthalmus*	Sca_ery	PEL, OMN, PHY, TOL	0.02			0.14	0.03		0.04	0.07	0.03	0.10
*Silurus glanis*	Sil_gla	BEN, PHY, TOL	0.14	0.74	1.03	0.56	0.24	2.84	0.29	1.92	0.24	1.49
*Squalius cephalus*	Squ_cep	PEL, EUR, OMN, POT, TOL	14.11	7.57	15.37	5.96	15.72	4.12	12.29	9.81	9.41	5.71
*Thymallus thymallus*	Thy_thy	PEL, RHE, INS, POT		0.47								
*Tinca tinca*	Tin_tin	BEN, OMN, PHY, TOL	0.04	0.82	0.16	0.25	0.17			0.13		
	Car_spp	BEN, OMN, PHY, TOL						0.32		0.03		0.09
*Carassius gibelio*							1.50		0.01		0.07	
*Carassius carassius*					0.01		0.88					
*Carassius auratus*							0.08					
	Leu_spp	RHE, OMN, POT		1.78		1.05		0.14				
*Leuciscus leuciscus*			1.95		0.07							
*Leuciscus idus*							0.03				0.01	
	Cypr_1	RHE, POT		11.83		12.89		5.68		3.90		2.87
*Parachondrostoma toxostoma*									0.01			
*Chondrostoma nasus*			10.15		1.16		7.10		2.06		2.92	
*Telestes souffia*			0.02		0.20		0.07		0.27		0.01	

Species groupings: BEN, benthic feeding; EUR, eurytopic (generalist); INS, insectivorous; OMN, omnivorous; PHY, phytophilic; POT, potamodromous (migratory movements in freshwater habitats); RHE, rheophilic species; TOL, tolerant to pollution species; PEL, pelagic species ().

The species richness assessed from TEF in spring 2016 was significantly lower (ANOVA, *P* < 0.001) than that obtained by eDNA at the five sites (13 to 24 species against 28 to 29 species, respectively). When the three TEF sampling occasions from summer 2015 to spring 2016 were pooled, the richness no longer differed between the two methods (19 to 27 species against 28 to 29 species, respectively; *P* > 0.05). When considering the six TEF sampling occasions (summer 2014 to spring 2016), TEF and eDNA sample richness values were very similar (23 to 31 species against 28 to 29 species, respectively; *P* > 0.05). Species accumulation curves (R software, vegan package, specaccum function; Oksanen *et al*., 2017) showed that the average species richness estimated from either a single campaign or three TEF campaigns was comparable among the five RSs when compared with the cumulative species richness for the full period of 2014–2016 (61.1%–65.4% and 84.4%–89.2% of the total number of species caught during the period 2014–2016, respectively).

**FIGURE 2:**
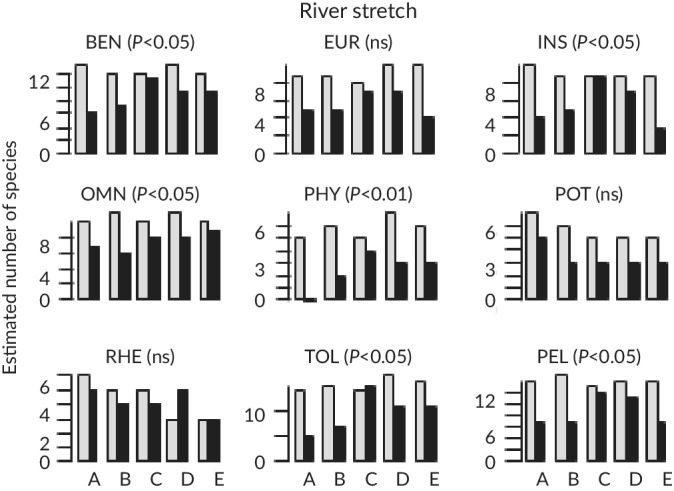
Comparison of trait‐based metrics expressed in number of species computed from eDNA (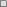) and traditional electro‐fishing (TEF; 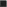) samples in the five river stretches (RS), A, B, C, D and E. Trait categories: BEN, benthic; EUR, eurytopic; INS, insectivorous; OMN, omnivorous; PHY, phytophilic; POT, potamodromous; RHE, rheophilic; TOL, tolerant; PEL pelagic. Significance of the differences between eDNA and TEF metrics are shown: *P <* 0.05; *P* < 0.01; ns, not significant (*P* > 0.05)

**FIGURE 3:**
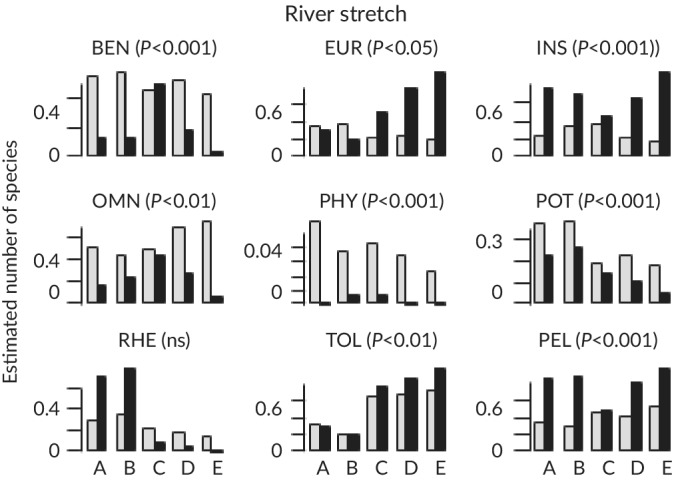
Comparison of trait‐based metrics expressed in relative number of individuals computed from eDNA (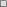) and traditional electro‐fishing (TEF; 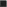 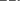) samples in the five river stretches (RS), A, B, C, D and E. Trait categories: BEN, benthic; EUR, eurytopic; INS, insectivorous; OMN, omnivorous; PHY, phytophilic; POT, potamodromous; RHE, rheophilic; TOL, tolerant; PEL pelagic. Significance of the differences between eDNA and TEF metrics are shown: ns, not significant (*P* > 0.05)

The four‐following richness‐based metrics were significantly lower for TEF samples from spring 2016 than those computed for eDNA samples (Figure 2): benthic (*P* < 0.05), insectivorous (*P* < 0.05), pelagic (*P* < 0.05) and phytophilic (*P* < 0.01) species. With increasing TEF sampling effort, these differences became non significant (*P* > 0.05). Among the metrics based on relative abundance (Figure 3), only the rheophilic species metric did not significantly differ between TEF samples from spring 2016 and eDNA samples (*P* > 0.05). Four TEF metrics had higher values than the eDNA‐based metrics: eurytopic (*P* < 0.05), insectivorous (*P* < 0.001), tolerant *(P* < 0.01) and pelagic *(P* < 0.001) species. The four other eDNA‐based metrics had higher values than the TEF‐based metrics: benthic *(P* < 0.001), omnivorous *(P* < 0.01), phytophilic *(P* < 0.001) and potamodromous *(P* < 0.001) species. The differences between eDNA‐ and TEF‐based metrics remained significant for six of the last eight metrics with increasing TEF sampling effort but not for eurytopic and potamodromous species.

### 3.3 Comparison between eDNA‐based and current ecological assessments

The association between the ecological assessment (ecological status class) obtained with eDNA‐10FI and the current ecological assessment was not significant (*χ* ^2^‐test = 2.14, *df* = 1, *P* > 0.05). When only the six metrics expressed in relative number of species or individuals were considered (eDNA‐6FI), the two assessments were significantly associated (*χ* ^2^‐test = 11.58, *df* = 1, *P* < 0.001). Among the 25 sites, 22 were correctly classified (88%) with the adapted eDNA‐based fish index: 12 with a good ecological status (ecological class higher than or equal to good) and 10 with a degraded status (ecological class lower than good).

**FIGURE 4:**
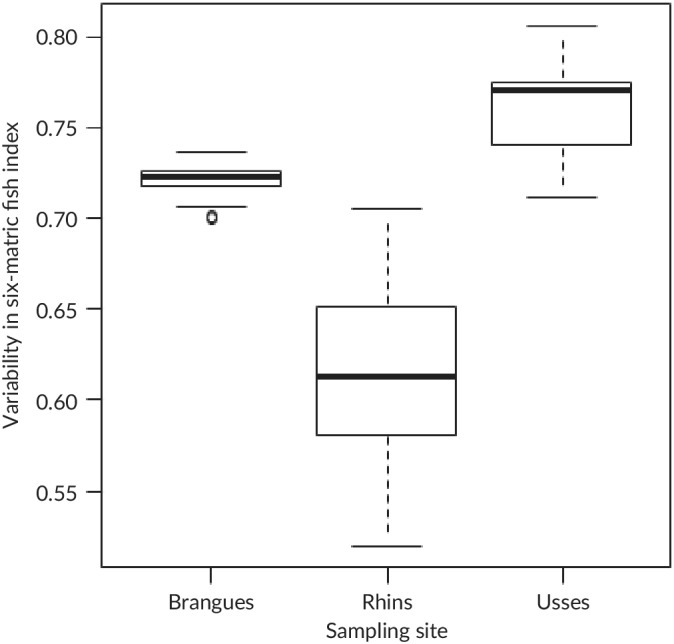
Boxplots (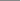, median value; 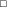, interquartile range; 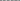, full range; , outliers) showing the variability in the eDNA‐adapted fish index (six metrics) computed at three sites (Brangues, Rhins and Usses) where 10 eDNA water samples were collected once. At each site, the eDNA‐based six‐metric fish index was computed for each of the 45 possible pairs of samples

The values of eDNA‐6FI computed for each of the 45 possible pairs of the ten eDNA samples varied from 0.70 to 0.74, 0.52 to 0.71 and 0.71 to 0.81 for the Brangues, Rhins and Les Usses Rivers, respectively (Figure 4); CV = 0.011, 0.0787 and 0.034, respectively. All 45 combinations were classified as having the same ecological status in the Brangues and Les Usses Rivers (good status). In the Rhine River, all the combinations were classified as having a moderate status except one (good status). The seasonal variability of eDNA‐6FI was low (Table 3) in the three studied river stretches (Ain, Jons and Tier). The ecological quality class remained unchanged for the six sampling dates except for at Jons in April 2016.

**TABLE 3:** Seasonal variability (October 2015 to August 2016) of the ecological assessment of three river sites using an eDNA‐based six metric fish index (eDNA‐6FI). Numerical values of the fish index and corresponding ecological quality classes (in parentheses) are provided. The official EU Water Framework Directive status of the corresponding water bodies from the Ain River, the upper Rhône at Jons and the Tier Rivers are good, moderate and moderate, respectively

	Ain River	Jons River	Tier River
October	0.75 (Good)	0.68 (Moderate)	0.66 (Moderate)
December	0.74 (Good)	0.69 (Moderate)	0.68 (Moderate)
February	0.76 (Good)	0.68 (Moderate)	0.59 (Moderate)
April	0.72 (Good)	0.65 (Good)	0.60 (Moderate)
June	0.74 (Good)	0.69 (Moderate)	0.64 (Moderate)
August	0.74 (Good)	0.73 (Moderate)	0.64 (Moderate)

## 4 DISCUSSION

### 4.1 WFD‐compliant national methods

Our review of the 25 current WFD‐compliant river fish indices shows that more than 80% of the total number of metrics considered are expressed in number of species or in relative abundance of individuals and are therefore compatible with eDNA‐based assessment methods. However, 14 out of 25 national indices also incorporate metrics based on population age or size structure, which cannot be computed from eDNA samples. Metrics based on age or size structure for one or more fish species are recommended to be included in any WFD‐compliant multi‐metric fish indices (EC, 2000), but this criterion was not included in the WFD compliance check of the national assessment methods (Pont, 2011). ECOSTAT mandates not considering such types of metrics when rational explanations are provided. Currently, none of the 25 WFD‐compliant intercalibrated methods include metrics based only on species richness or relative abundance, without also including some metric based on age or size structure. This last metric could be important to better assess low‐diversity brooks. It might be worth knowing how much weight these age or size structure metrics really have on classification compared with the richness–relative abundance metrics.

All the currently used indices including age or size structure metrics cannot be directly calculated from eDNA samples. The most commonly used other metrics in current fish indices can be computed but as they are expressed in number of species, relative abundance or biomass (Pont *et al*., 2018; Thomsen *et al*., 2012). Consequently, an adaptation of these methods to allow the assessment of the ecological status of rivers with eDNA‐based methods seems conceivable. Among the options proposed by Hering *et al*. (2018) to include eDNA‐based methods into WFD ecological status assessment, option 2 (new–adapted assessment methods) seems the most realistic. However, the situation will vary depending on the member state and its currently used method.

### 4.2 Representativeness of eDNA‐based metrics

Five of the nine ecological guild richness‐based metrics computed from eDNA samples had higher values than those obtained from TEF samples collected in spring 2016 (benthic, insectivorous, phytophilic, tolerant and pelagic species). After summing the number of species caught during all the available TEF sampling campaigns (2014–2016), the species trait richness obtained by the two methods were comparable. This result is consistent with results of previous studies (Civade *et al*., 2016; Valentini *et al*., 2016; Wilcox *et al*., 2016). eDNA samples produce a more comprehensive species list than TEF samples and TEF sampling effort needs to be strongly increased (either over a long period of time or *via* multiple samplings in one year) to capture a species richness equivalent to that obtained with a single eDNA sampling (Pont *et al*., 2018). Traditional methods in large rivers are inappropriate to provide a comprehensive species list with limited sampling effort (Eros *et al*., 2017; Zajicek & Wolter, 2018). An increase in the number of phytophilic species obtained with eDNA samples was evident in all RSs. All species belonging to this guild were rare in our dataset (less than 1% of the total abundance in TEF) and eDNA seems to be more appropriate than TEF to evaluate their diversity with limited sampling effort.

In contrast to richness‐based metrics, metrics based on relative abundance were significantly different between TEF and eDNA samples, regardless of the sampling effort. Furthermore, depending on the metric, they were either higher or lower. Previous works demonstrated moderate correlations between the number of eDNA copies and species abundance or biomass (Evans *et al*., 2016; Li *et al*., 2019; Pont *et al*., 2018; Thomsen *et al*., 2012). In deep rivers, where TEF can be performed only along banks due to high sampling inefficiency elsewhere, sub‐surface species are overestimated by TEF (Zajicek & Wolter, 2018) in comparison with benthic species. Pont *et al*. (2018) argued that such bias can partly explain the moderate correlation between TEF and eDNA samples in large rivers. The present results confirmed this statement. The relative abundance of pelagic species was higher with TEF, whereas the benthic species were better represented by eDNA. A similar situation was observed for eurytopic, insectivorous and tolerant species; *i.e*., all metrics where *Alburnus alburnus* (L. 1758) represented a large part of the individuals caught by TEF. This species is known to be overrepresented in shoreline TEF samples, sometimes to the point that *A. alburnus* must be removed from statistical analyses to be able to perceive fine changes in the composition of fish assemblages between different river stretches (*e.g*., Eros *et al*., 2017). In our dataset, *A. alburnus* represented 69.8% of the TEF total catch by individuals compared with only 6% of the total number of DNA copies. In contrast, the higher relative abundance of potamodromous species in eDNA samples is of great interest for ecological assessment as these species and the corresponding metric are sensitive to connectivity disruption.

Phytophilic species are also of great interest as they include typical floodplain specialist species (Schomaker & Wolter, 2011), which are sensitive to habitat degradation such as channelisation or disruption of lateral connectivity. These two metrics tended to decrease from upstream to downstream, reflecting a higher degree of artificicality of the water bodies (Figure 2). Conversely, eurytopic, omnivorous and tolerant species were relatively more abundant in the downstream RSs, with a maximum in RS E, which was the most artificialised RS (concrete channel). More generally, the evenness computed from eDNA samples is higher than that computed from TEF samples (Pont *et al*., 2018). Based on the present results, we conclude that eDNA is likely to provide a better overview of the functional diversity of fish assemblages in large rivers than TEF. The use of eDNA‐based methods could therefore subsequently improve the sensitivity of the currently used trait‐based metrics to the different types of anthropogenic pressures.

### 4.3 Comparison between eDNA‐based and current ecological assessments

Environmental DNA‐6FI demonstrates the potential of an eDNA‐based method for WFD ecological assessment (88% of the sites were correctly classified). However, this preliminary result also highlights the need to adapt the current TEF‐based WFD assessment methods to such new sampling techniques. Even for richness‐based metrics, metrics based on absolute values rather than relative ones must be avoided in the eDNA‐6FI to obtain an ecological assessment comparable with the current one. Based on our previous comparison of the different types of metrics (see above), this result is probably related to inadequate sampling effort with classical electrofishing techniques. Furthermore, the associated uncertainties estimated at the three sites with eDNA‐6FI were always lower than 0.1, which is quite low compared with the uncertainties associated with classic or predictive IBIs (Dolph *et al*., 2010; Logez *et al*., 2019; Marzin *et al*., 2014; Schoolmaster *et al*., 2012). When reducing the uncertainty associated with sampling, a better signal to noise ratio (Hughes *et al*., 1998) can be expected. The seasonal variability of eDNA‐6FI is also limited, but this does not mean that eDNA samples are not able to detect seasonal changes in dominant fish species (Milhau *et al*., 2019).

Independent of contamination or structural errors during sampling or laboratory work, a false positive can be related to the presence of DNA in samples due to downstream eDNA transportation (Civade *et al*., 2016; Hering *et al*., 2018; Pont *et al*., 2018). This was the case for two salmonid species (*C. lavaretus* and *S. alpinus*) inhabiting upstream lakes flowing into the Rhône River. More broadly, DNA transport from upstream areas can also be related to inflows from tributaries or backwaters. In this case, species may be present in the main watercourse, but their relative abundances may be artificially increased when the sample is collected near a confluence. The ecological assessment of the main watercourse may thus be biased. The presence of eDNA from wastewater release or from fish farms may also alter ecological assessment. It is therefore recommended to avoid sampling locations immediately downstream from cities and fish farms or too close to a confluence with a tributary or backwaters. The importance of the flow of these inputs relative to that of the main watercourse (dilution) must be considered before choosing the eDNA sampling point. In the case of fish, the release of eDNA by dead animals is probably limited, except in very small streams, contamination *via* dead bait introduced by anglers or after mass mortality.

In conclusion, our first adaptation of an existing fish index provides encouraging results, showing good comparability with the current ecological assessment of water bodies. This first result must be considered a proof of concept and the development of an eDNA‐based assessment method for fish in rivers should be based on a much more extensive set of sites, covering the full range of ecological conditions and including extreme situations (very good and very bad ecological statuses). Additionally, to compare eDNA and conventional assessment methods, site selection should focus on long time series of electrofishing surveys to limit TEF bias regarding species richness underestimation.

Adapting reference conditions for eDNA‐based methods is probably not required when the reference conditions are defined based on a combined examination of historical–present data and expert judgement, unless rare species, which are better represented in eDNA samples than in TEF samples, prove to be insufficiently considered. When the indices and metrics are calibrated using an actual reference dataset (*i.e*., independent reference sites), we recommend resampling the reference sites using the eDNA method to obtain an appropriate definition of reference conditions for the different metrics considered, whether based on taxonomy or ecological or biological traits. The observed fish assemblages should then be calibrated against reference conditions where the rare species and trait diversity are better estimated. It would also be necessary to ensure to which point eDNA could lead to heightened detection of non‐native species from reference sites. We hypothesise lower uncertainties associated with the index values because of the good reproducibility of eDNA samples. In such a case, we could expect a reduction in the signal‐to‐noise ratio (Hughes *et al*., 1998) and improvement in pressure–effect relationships. A comparison of the uncertainties associated with conventional and eDNA methods would allow us to test this assumption.

In rivers, eDNA‐based assessment methods would also differ from conventional methods due to their spatial representativeness. Using a model based on experimental and observational studies estimating eDNA detection distance in rivers, Pont *et al*. (2018) showed that eDNA has a sedimentation rate comparable with that observed for fine particulate organic matter. Similarly, its vertical transfer from the water column to the riverbed (velocity deposition) is highly dependent on the mean velocity and mean depth of the water body (Cushing *et al*., 1993; Minshall *et al*., 2000). In small shallow streams, the eDNA detection distance is on the order of magnitude of km. However, for medium and large rivers, the downstream transportation distance of a MOTU can vary from a 10 km to 100 km. Then, in a small stream, the eDNA transportation distance is comparable with the scale at which traditional sampling techniques are performed. As the river size increases, eDNA is conveyed farther downstream and produces a more spatially integrated measure of biodiversity (Pont *et al*., 2018), which becomes increasing decoupled from local habitats (Deiner & Altermatt, 2014; Deiner *et al*., 2016) and spatial habitat heterogeneity at the reach scale, but perhaps better represents the situation at the water‐body scale.

Fish eDNA‐based methods appear to be a promising tool for river assessment in Europe, particularly for large rivers, where current fish sampling techniques have known shortcomings. Special attention should be paid to small streams to test the ability to assess such low fish diversity environment without age or compared with size‐based metric. In most cases, the current fish assessment methods need to be adapted and further investigations are required before moving to the application phase. Additionally, the spatial representativeness of fish eDNA samples in rivers is different from that of traditional fishing methods, which implies that the option of “only DNA‐based identification” (option 1; Hering *et al*., 2018) is not feasible.

Within a few years, we can confidently expect the availability of fish eDNA‐adapted assessment methods with high sensitivity, better reproducibility and lower associated uncertainties. A roadmap for the implementation of fish DNA‐based method in the WFD should include a survey of rivers covering the different ecoregions of Europe, the main environmental gradients (climate, size and slope of the river) and sites characterised by anthropogenic disturbances of varying intensity and types (water quality, hydromorphological disturbances, disruption of connectivity *etc*). Based on our previous experience (Pont *et al*., 2006), 4000 to 5000 sampling sites would be needed to define baseline conditions and test the sensitivity of fish eDNA‐based method to anthropogenic pressures (pressure–effect relationships).

## AUTHOR CONTRIBUTIONS

M.R. and T.D. designed the study. T.D., P.J. and M.R. collected the eDNA samples in the field. T.D., P.J. and A.V. conducted the laboratory and bio‐informatics analyses. A.M. prepared and provided the electrofishing survey data. O.D. provided the environmental data needed to perform the fish‐based ecological assessment method. D.P. performed the statistical analysis, prepared the figures and wrote most of the manuscript, with significant contributions from all the other authors.
